# Exploring the Conformers of an Organic Molecule on a Metal Cluster with Bayesian Optimization

**DOI:** 10.1021/acs.jcim.2c01120

**Published:** 2023-01-16

**Authors:** Lincan Fang, Xiaomi Guo, Milica Todorović, Patrick Rinke, Xi Chen

**Affiliations:** † Department of Applied Physics, Aalto University, 00076AALTO, Finland; ‡ State Key Laboratory of Low Dimensional Quantum Physics and Department of Physics, Tsinghua University, 100084Beijing, China; ¶ Department of Mechanical and Materials Engineering, University of Turku, FI-20014Turku, Finland

## Abstract

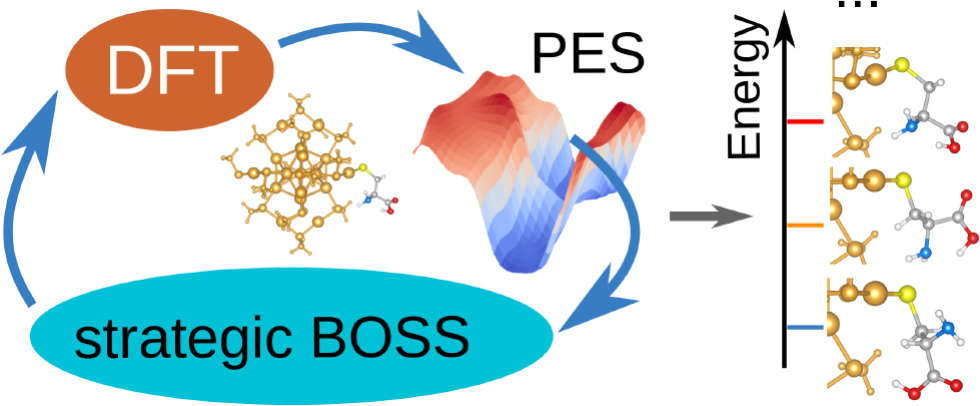

Finding low-energy conformers of organic molecules is a complex problem due to the flexibilities of the molecules and the high dimensionality of the search space. When such molecules are on nanoclusters, the search complexity is exacerbated by constraints imposed by the presence of the cluster and other surrounding molecules. To address this challenge, we modified our previously developed active learning molecular conformer search method based on Bayesian optimization and density functional theory. Especially, we have developed and tested strategies to avoid steric clashes between a molecule and a cluster. In this work, we chose a cysteine molecule on a well-studied gold–thiolate cluster as a model system to test and demonstrate our method. We found that cysteine conformers in a cluster inherit the hydrogen bond types from isolated conformers. However, the energy rankings and spacings between the conformers are reordered.

## Introduction

Organic–inorganic hybrid systems composed of a metal core and organic molecules have become increasingly important in modern nanotechnology. The physical and chemical properties of the organic–inorganic heterostructures are highly tunable by, e.g., engineering the metal core, the molecules, and the molecule–metal interfaces. Their potential applications include catalysis,^1^ molecular sensors,^2^ bioimaging,^3^ and nanomedicine.^4^ However, hybrid material design and optimization remain fundamentally challenging because the desired properties often depend on the microscopic structure of the system, which is typically unknown.

Computational simulations, especially density functional theory (DFT), have proven essential for microscopic structure determinations of hybrid systems as they can accurately predict the structure on an atomic level, which experiments often cannot resolve. For a given molecule–cluster system, atomic models of the metal part and metal–molecule interface can be built from experimental data (e.g., electron microscopy),^5^ previous reported crystal structures,^6^ chemical intuition,^7^ or data-driven methods.^8^ However, the configurations of adsorbed molecules are difficult to determine.

Finding low-energy molecular conformers in the gas phase is already a challenging problem. Organic molecules are usually flexible and have a high-dimensional structural search space with many local minima. In addition, to accurately predict structures and energies of molecular conformers, costly quantum mechanical accuracy is required.^9^ To address this conformer-search challenge, a variety of methods and tools such as systemic methods,^10^ stochastic methods,^11,12^ hierarchical methods,^13,14^ and machine learning techniques^15−18^ have been developed for molecular conformer search.

In recent years, Bayesian Optimization in combination with DFT has been widely applied to molecules and materials for structure search and property optimization.^19−21^ In our recent work, we developed an active machine learning procedure for molecular conformer identification and ranking. We first fix the bond lengths and angles and choose the molecular dihedral angles as features to reduce the dimension of the search space. Then, we employ the Bayesian Optimization Structure Search (BOSS) code^19,22^ to build a surrogate potential energy surface (PES) model in the reduced dimensions with iterative Bayesian Optimization (BO) sampling.^16^ After the model converges, we extract the local minima from the surrogate PES and optimize the corresponding structures with DFT before adding free-energy contributions and quantum chemistry corrections. More details can be found in ref (16).

We have validated the accuracy and efficiency of our procedure on cysteine, serine, tryptophan, and aspartic acid.^16^ However, the BOSS conformer search was not directly applicable to molecular conformers on a cluster, because close molecule–cluster contact or steric clashes could not be handled. If atoms in a structure come too close, the DFT energy diverges or the DFT code returns an error, which means that no data point is returned to continue the iterative algorithm. This presents a problem in active learning. It leads to large model uncertainties and causes the acquisition function to repeatedly query the unphysical region of the search space. In this work, we developed strategies to address steric clashes in active learning by constructing different solutions in the regions where energy data could not be evaluated.

**Figure 1:**
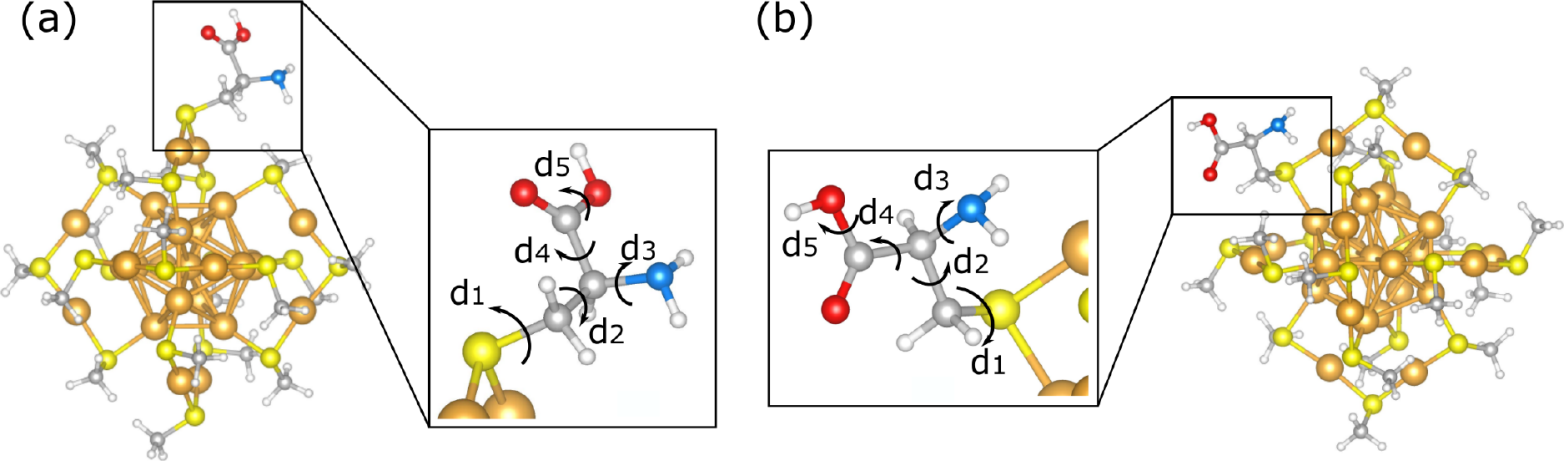
Ball–stick model of cysteine on Au_25_(SCH_3_)_17_ cluster: (a) system A and (b) system B. Gold color is used for gold atoms, red for oxygen, white for hydrogen, gray for carbon, blue for nitrogen, and yellow for sulfur. *d*_1_, *d*_2_, *d*_3_, *d*_4_, and *d*_5_ label the five dihedral angles of cysteine that we use to define our search space.

We used a cysteine molecule adsorbed on a well-studied thiolate-protected Au_25_ cluster^6,23^ as a model system to test and demonstrate our method. We chose the system for several reasons. First, it offers us an ideal situation of cysteine in a complicated, confined environment. Second, the cluster contains a Au_13_ icosahedral core, protected by six SCH_3_-Au-SCH_3_-Au-SCH_3_ V-shaped staples.^6,23^ Consequently, cysteine can adsorb at two inequivalent S sites: one on the top of the V-shape staple, and the other on the side, as shown in Figure 1a and b. This allows us to study how the different surrounding environments affect the structures of cysteine. Third, we can compare to cysteine conformers in the gas phase from our previous study^16^ to elucidate confinement and proximity effects.

In brief, we used a cysteine on a gold–thiolate cluster as a model system to develop and test strategies for avoiding steric clashes in BOSS active learning in the Methods section. In the Results and Discussion section, we compared the different strategies and applied the optimal one to investigate how the gold–thiolate cluster affects the structures and energy rankings of the cysteine conformers.

## Methods

Our procedure contains three steps: (i) system preparation, (ii) BOSS strategic structure search, and (iii) refinement, in analogy with the procedure we had developed for the molecular conformer search in the gas phase.^16^

In step (i), we first build an atomic model of the Au_25_(SCH_3_)_17_(Cys) (Cys: cysteine) by replacing one SCH_3_ in the well-studied Au_25_(SCH_3_)_18_ cluster with a deprotonated cysteine molecule. SCH_3_ was chosen here to reduce the computational cost and the complexity. We optimized this structure with DFT and set the total energy of the optimized structure as the energy reference 0 eV for the BOSS sampling in the next step. Since we are interested in searching the configurations of cysteine here, we adopt the “building block” concept.^19,24^ Except for the atoms in cysteine, all other atoms are constrained during the machine learning procedure. As in our previous work,^16^ we choose dihedral angles as the most informative degrees of freedom to describe the different cysteine configurations and refer to the dihedral angle space as *phase space*. The bond lengths and angles are fixed at their optimized values during BOSS sampling, because they are relatively rigid. Cysteine has two nonequivalent bonding sites on the Au_25_(SCH_3_)_17_ cluster as illustrated in Figure 1a and b. We consider both of them and refer to them as system A and system B in the rest of the paper (Figure 1).

In step (ii), we employ BOSS to actively learn the PES of the system by BO iterative sampling. Each sample point contains dihedral angles *d*_*i*_ (*i* = 1, 2, 3, 4, 5) of cysteine (Figure 1) and the corresponding energy of Au_25_(SCH_3_)_17_(Cys).

For small molecules in the gas phase, each dihedral angle contributes 360° to the full phase space (discounting possible rotational symmetries). This angular range might be restricted for a molecule on a cluster. Configurations that lead to steric clashes typically have a high energy and are therefore not important in the search for global minima, but they present a challenge to active learning. DFT calculations either crash for steric clashes or they return unfavorably high energies. The inability to sample a region of the search space leads to large uncertainties in the surrogate model, promoting explorative sampling and causing the active learning algorithm to repeatedly query this, ultimately irrelevant, part of the search space. Even if a DFT computation could proceed, including very high energies into the energy landscape produces a large model variance. This makes the model less accurate in the low-energy PES regions of interest, which then requires more sampling to refine.

To address these technical challenges, we devised and tested three different strategies to prevent sampling nonphysical structures. In strategy **i**, we try to identify a continuous region in phase space that is free of steric clashes. BOSS then samples only in this restricted phase space. In strategy **ii**, we define a “safe minimum distance” *D*_0_ between any two atoms in the system to preselect physically meaningful structures. If the shortest distance between the cysteine and other atoms is longer than *D*_0_, the energy of the structure will be calculated by DFT, otherwise a constant energy will be returned. In strategy **iii**, we use a logarithmic energy transformation to suppress high energy regions and apply an energy penalty to nonphysical structures for which DFT cannot return an energy. We discuss the details of the strategies and their advantages and limitations below.

After the PES has been learned sufficiently well, in step (iii), we use the BOSS postprocessing routines to analyze the surrogate PES and extract the local minima configurations and related structures. In this step, we relax all degrees of freedom in the systems to refine the structures and energies.

The all-electron code FHI-aims^25−27^ was applied for all DFT calculations. “Tight” numerical settings, “tier 2” basis sets for S, C, O, N, H, and the “tier 1” set for Au were used throughout. Following our previous work,^16^ we used the Perdew–Burke–Ernzerhof (PBE) functional^28^ with many-body dispersion corrections^29^ in all DFT calculations. For structure optimizations, the geometry was considered to be converged when the maximum residual force (fmax) was below 0.01 eV/Å.

During the sampling, BOSS employed Gaussian process (GP) models to fit a surrogate PES to the data points, and then refined it by acquiring more data points at locations that minimize the exploratory lower confidence bound (eLCB) acquisition function. We applied a “rbf” kernel for the nonperiodic *d*_*i*_ in strategy **i** and a “stpd” kernel for the periodic *d*_*i*_ in strategies **ii** and **iii**.

In the interest of open science,^30,31^ we make the results of all relevant calculations freely available on the Novel Materials Discovery (NOMAD) repository.^32^

### Sampling in the Limited Phase Space

The most intuitive way to avoid steric clashes is to exclude those regions of phase space from sampling. To investigate whether such a “safe” region exists for our systems, we randomly generated 100,000 *d*_*i*_ (*i* = 1, 2, 3, 4, 5) structures for both systems A and B. Then, we calculated the shortest atomic pair distance *D*_*min*_ between cysteine and Au_25_(SCH_3_)_17_ in each structure and plotted *D*_*min*_ against *d*_*i*_ in Figures S1–S3. If we limit the sampling range of *d*_1_, there is a region where the cysteine and cluster will not become too close (Figure S1). However, we cannot exclude all the structures with steric clashes by restricting *d*_2_, *d*_3_, *d*_4_, or *d*_5_ (Figures S2–S3). The reason is that *d*_1_ is the closest rotational bond to the cluster, thus determining the overall structure.

**Figure 2:**
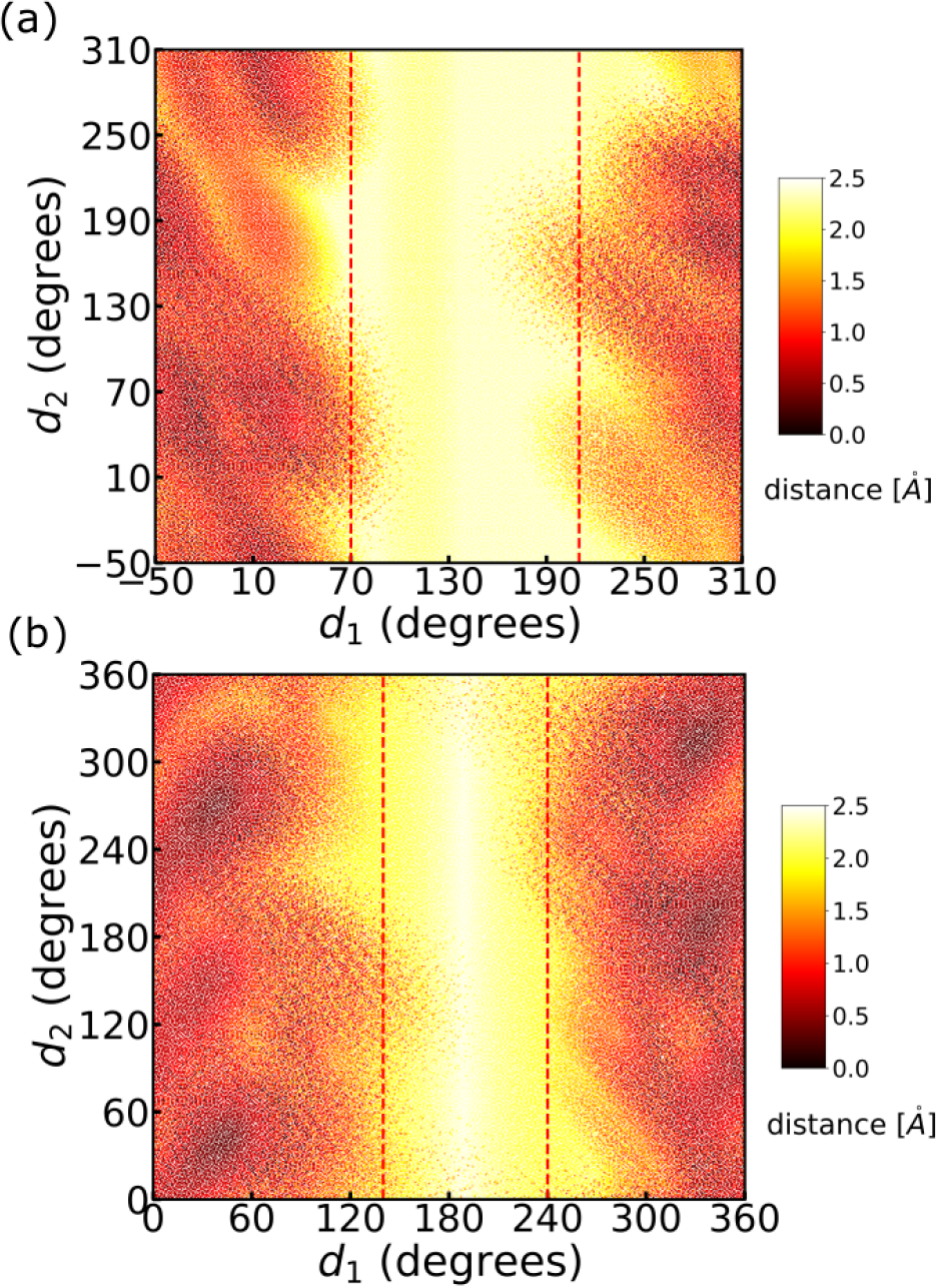
Two-dimensional (*d*_1_ and *d*_2_) maps of the shortest atomic pair distance *D*_*min*_ between cysteine and the cluster for (a) system A and (b) system B. In the plots, a light color means the structures have large *D*_*min*_, while a dark color means the structures have small *D*_*min*_, where the steric clash may happen. Strategy **i** only sampled *d*_1_ between dashed red lines.

Figure 2a and b presents the *d*_1_–*d*_2_ 2-D distribution of *D*_*min*_ for systems A and B. The panels suggest that we can obtain a “safe” sample region by restricting *d*_1_ to [70°, 210°] for system A or [140°, 240°] for system B. In such a region, 99.7% of the structures have *D*_*min*_ > 1.0 Å; *d*_1_ has a narrower “safe” region in system B than in system A due to the more restricted local environment. This strategy is easy to apply and very unlikely to sample the nonphysically meaningful structures, but it may miss parts of phase space that correspond to low-energy structures, such as the yellow areas to the left or the right of the red dashed region in Figure 2a and b.

### Safe Distance Selection

In strategy **ii**, we address the clash problem by introducing a safe distance *D*_0_ to classify “safe” and “unsafe” structures and treat them differently. If the shortest atomic distance *D*_*min*_ between the cysteine and the Au_25_(SCH_3_)_17_ is larger than *D*_0_, the structure will be identified as a physically meaningful one, and its energy will be its DFT energy *E*. Here, we chose *D*_0_ equal to 1.4 Å, which lies around the typical bond length in organic molecules. In contrast, if *D*_*min*_ is smaller than *D*_0_, the structure will be considered nonphysical, and no DFT calculation will be performed. Instead, a constant energy *E*_0_ will be assigned to the structure. The energy *E*_*new*_ for updating the GP in BOSS sampling is
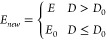
1

In our tests, we tried constant and harmonic potentials for *E*_0_. The agreement between the surrogate BOSS energy and DFT single-point energies for selected structures did not improve for harmonic potentials. Therefore, we discuss only constant potentials in this work. We tested *E*_0_ = 2.0, 4.0, and 6.0 eV and found that the sampled structures have a similar energy distribution for system A (Figure S4). Therefore, we only present the results for *E*_0_ = 6.0 eV here.

The advantage of strategy **ii** is its capacity to sample the entire phase space without performing DFT calculations for nonphysical structures. The disadvantages are that the surrogate PES is not accurate for structures with *D*_*min*_ < *D*_0_, and the energy–structure relation is discontinuous around *D*_0_ which could lead to suboptimal surrogate PES model fits. Since a structure with *D*_*min*_ smaller or close to *D*_0_ typically has a high energy, these disadvantages should not affect the low-energy PES prediction.

### Energy Transformation

In strategy **iii**, we introduce an energy cutoff *E*_*cut*_. For a structure with a high DFT energy *E* ≥ *E*_*cut*_, we use a logarithmic energy transformation to attenuate the high energy of the nonphysical structure during BOSS sampling. In addition, for the structures where DFT simulations fail, we apply a penalty energy *E*_*p*_ to update the GP during BOSS sampling.
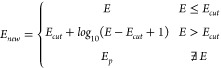
2We adopted *E*_*cut*_ = 2.0 eV and *E*_*p*_ = 4.5 eV in this work. Using *E*_*cut*_ = 1.0 or 3.0 eV did not change the main feature of *E*_*new*_ distribution (Figure S5). Strategy **iii** ensures a full phase space search and does not change the energy order of the structures where DFT calculations complete successfully. We employ smooth energy attenuation to bridge the landscape between high but chemically valid energies and the artificial energy penalty values and also to avoid introducing unphysical and sharp features into the model. The limitation is that the PES in the high-energy region is inaccurate, but this should not affect our low-energy structure search.

## Results and Discussion

We first monitor PES model convergence for the three strategies employed. Since our previous study on the 5-D or 6-D PES of amino acids showed that 1000 iterations are sufficient for learning their low-energy PES,^16^ we sample the PES of system A with 1000 BOSS iterations. The hyperparameters length scales for BOSS GP fitting stabilized around 600, 600, and 800 iterations for strategies **i**, **ii**, and **iii**, as shown in Figure S6. Figure S7 illustrates the refinement of the predicted global minimum with iterative configurational sampling for the three strategies. Both the energy and the dihedral angles of the predicted global minimum conformer converged around iteration 200 for strategy **iii**, while the energy and the dihedral angles of the predicted global minimum converged around iteration 800 for using strategy **i** and strategy **ii**. From this analysis, we can conclude that our surrogate PES was sufficiently converged with 1000 iterations.

After 1000 BOSS iterations, we extracted the local minima locations and their related structures from the surrogate PES and refined the structures by DFT optimization. Then, we purged duplicate structures and only kept unique configurations (Δ*d*_*max*_ > 10° and Δ*E* > 0.0057 eV). Using strategies **i**, **ii**, and **iii**, we finally obtained 37, 45, and 39 unique configurations within energy windows of 0.40, 0.68, and 0.63 eV from the global minimum, respectively.

**Figure 3:**
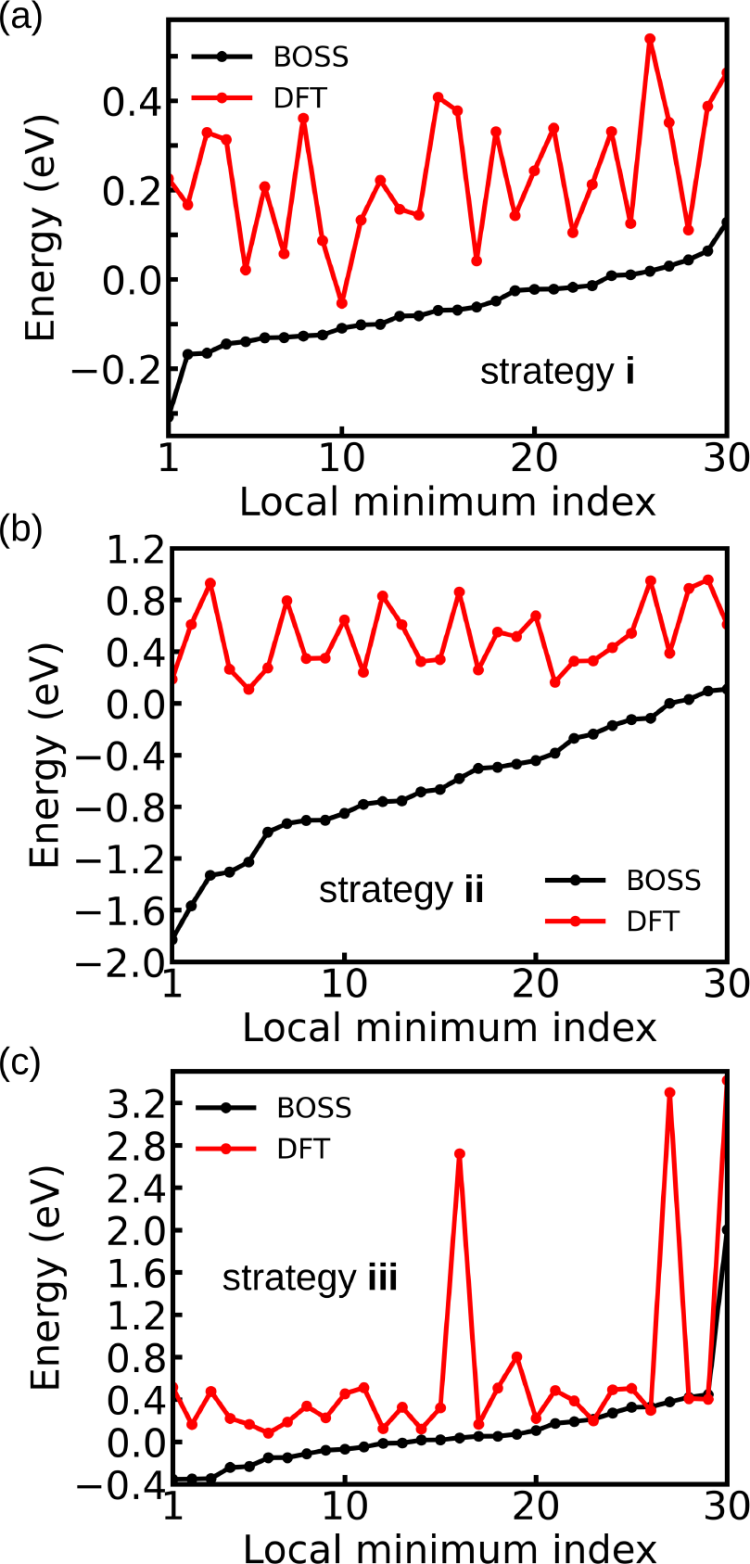
BOSS-predicted and DFT single-point energies of the 30 lowest-energy structures of system A with (a) strategy **i**, (b) strategy **ii**, and (c) strategy **iii**. The DFT energy of the atomic model we obtained in step (i) for the BOSS sampling was set to 0 eV.

We evaluated the strategies from two aspects: the accuracy of the surrogate PES and the energies of the final local minima structures we obtained. We picked the 30 lowest-energy structures after DFT relaxation for the evaluation. The accuracy of the surrogate PES is measured by the differences between the BOSS-predicted energies and the DFT single-point energies of the 30 structures before optimization. Figure 3a–c show that the DFT energy is generally higher than the BOSS-predicted energy for a given structure. The mean differences are 0.29, 1.14, and 0.52 eV for strategies **i**–**iii**. A smaller difference between the BOSS-predicted energy and the DFT single-point energy indicates that the surrogate PES is more accurate. From this perspective, strategies **i** and **iii** are better than strategy **ii**.

Systematic overestimation of BOSS minima values over DFT indicates a sharp variation of energy across the many peaks and minima in the interpolated surrogate model, but this is not relevant in our workflow. It is more important to retrieve the locations of the energy basins in the global landscape, which serve to initiate further local optimizations and compute local minima conformers.

**Figure 4:**
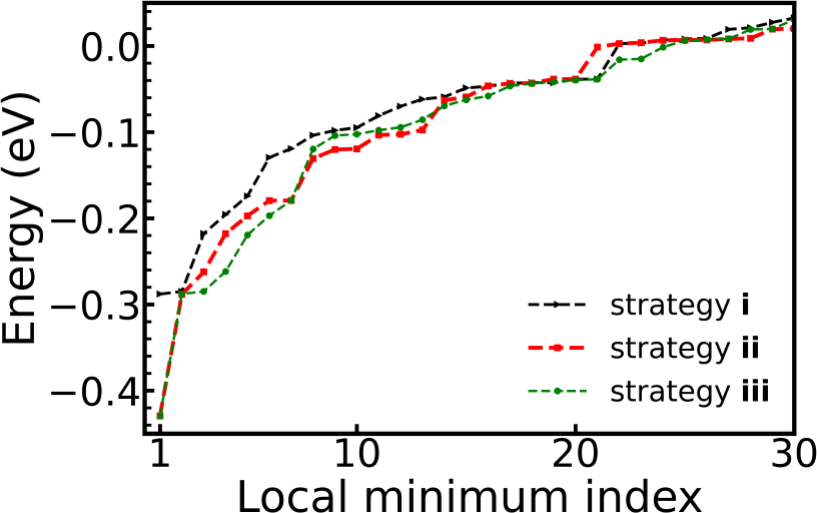
Relative energies of the 30 lowest-energy structures of system A after DFT optimization and removing duplicates. The DFT energy of the atomic model we obtained in step (i) for the BOSS sampling was set to 0 eV.

Figure 4 shows the energies of the 30 lowest-energy structures after DFT structure optimization. Overall the curves generated by strategy **ii** and strategy **iii** are very close to each other, suggesting strategies **ii** and **iii** have obtained very similar local minima structures. In the higher energy region, the three curves are almost on top of each other, indicating the three strategies found the same structures in this energy region. However, in the low-energy region, the green curve lies below the other two, because strategy **iii** found more stable structures.

Strategy **i** missed the global minimum structure, while strategies **ii** and **iii** succeeded in finding it. This is because system A has low-energy regions which cannot be sampled in strategy **i** (i.e., any yellow area to the left or the right of the red dashed region in Figure 2). Unfortunately, the global minimum of system A falls into such an unsampled low-energy region. Overall, strategy **iii** exhibits the best performance, and we chose strategy **iii** to study system B.

Next, we present the results of an active learning structure search for systems A and B. We obtained 39 unique local minima structures for system A and 31 unique local minima structures for system B. These structures have the relative energy within 0.63 eV from global minimum in system A and within 0.40 eV in system B. Similar to the isolated cysteine molecule, internal hydrogen bonds are commonly formed in the low-energy structures of adsorbed cysteine. If we use a cutoff of *r*_*HB*_ = 3.5 Å to define a hydrogen bond, all the local minima structures within a relative energy of 0.34 eV from global minimum in system A and 0.35 eV in system B contain at least one intramolecular hydrogen bond in cysteine.

**Figure 5:**
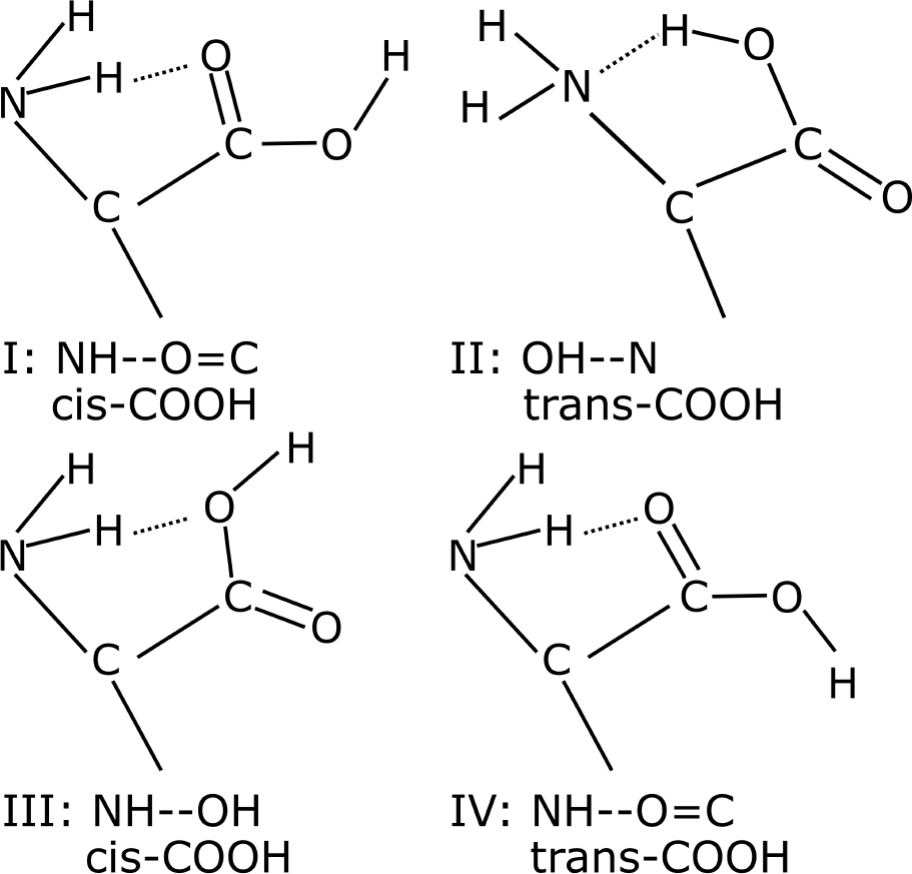
Types of hydrogen bonds between the amino group and the carboxyl group in cysteine.

We assess how Au_25_(SCH_3_)_17_ affects the configurations of cysteine and compare them to the isolated cysteine conformers. We choose the 11 cysteine conformers (Ia, Ib, I′b, IIa, IIb, IIc, III_α_a, III_α_b, III_α_c, III_β_b, III_β_c), identified in ref (33) and our previous work ref (16), as the reference structures for comparison. The conformers were named I, II, and III depending on the type of hydrogen bond (Figure 5) and as a, b, and c according to the configuration of the −CH_2_SH side chain.

The similarity of the two structures *a* and *b* can be measured with the following similarity index
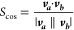
3Here, *v*_*a*_ and *v*_*b*_ are the vectors [cos(*d*_2_), cos(*d*_3_), cos(*d*_4_), cos(*d*_5_)] of the two structures (the definition of *d*_*i*_ is shown in Figure 1). Since *d*_1_ has an ambiguous meaning among the molecule conformers and systems A and (the H atom binding to the S atom in cysteine molecule was replaced by a gold atom for the adsorbed molecule), we did not include *d*_1_ in the similarity calculation. *S*_*cos*_ lies in the interval [−1,1]. The higher the value of *S*_*cos*_ is, the more similar the two structures are.

**Figure 6:**
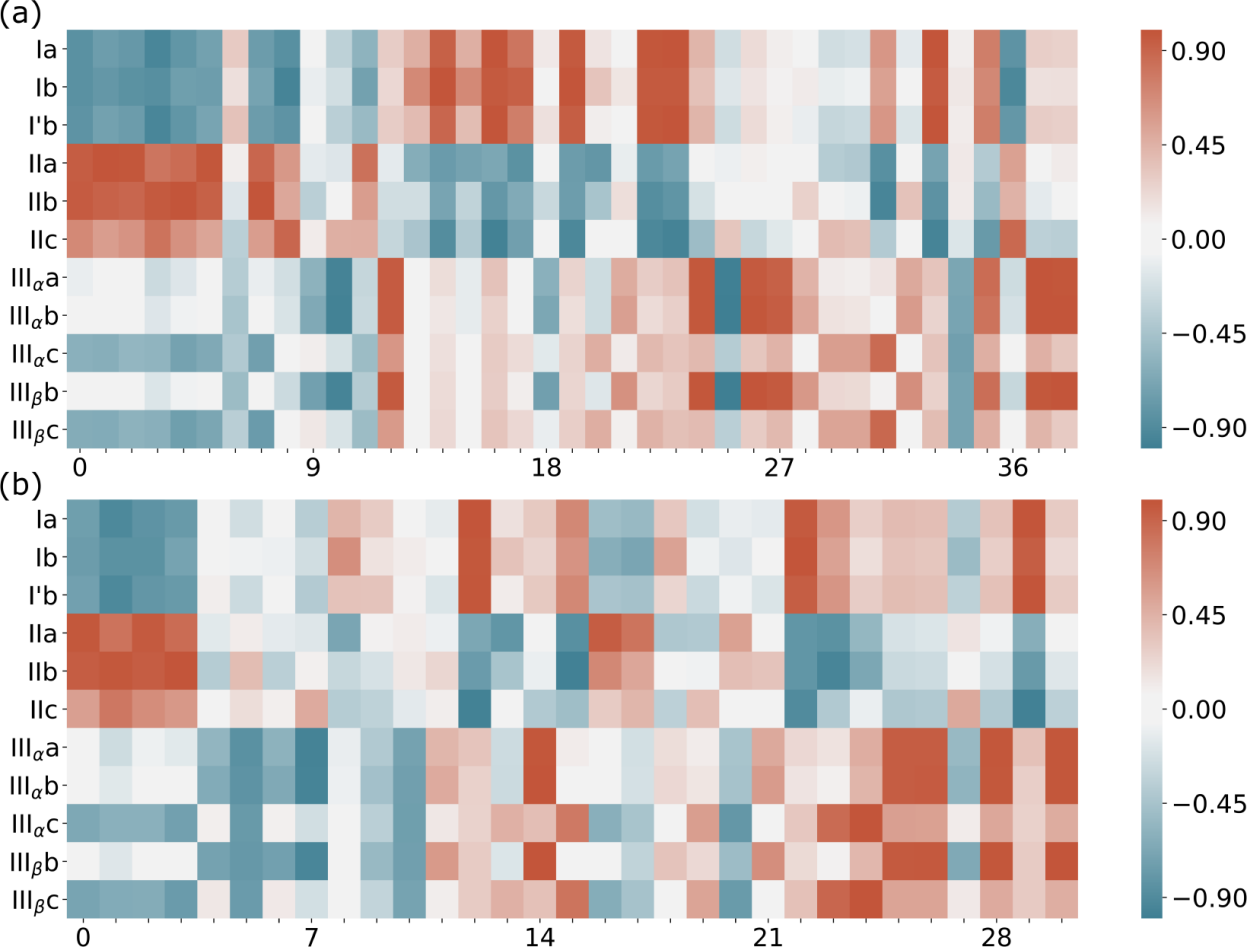
Similarity *S*_*cos*_ between the 11 reference cysteine conformers and the cysteine in the local minimum structures of (a) system A and (b) system B. The *y*-axis represents the 11 references; the *x*-axis lists the local minima structures of system A or system B in the increasing order of energy.

The *S*_*cos*_ of the cysteine configurations in systems A and B are plotted against the reference cysteine conformers in Figure 6a and b. Red colors indicate high similarity and blue low similarity. The red horizontal regions of high similarity in Figure 6 suggest that cysteine may form types I, II, and III hydrogen bonds when it adsorbs on Au_25_(SCH_3_)_17_. However, there are local minima structures in systems A and B where the configurations of cysteine are not similar to any reference molecular conformers. After analysis, we found that most of these structures form a new type of hydrogen bond (NH–O=C hydrogen bond, trans-COOH configuration), which we named type IV (Figure 5).

**Figure 7:**
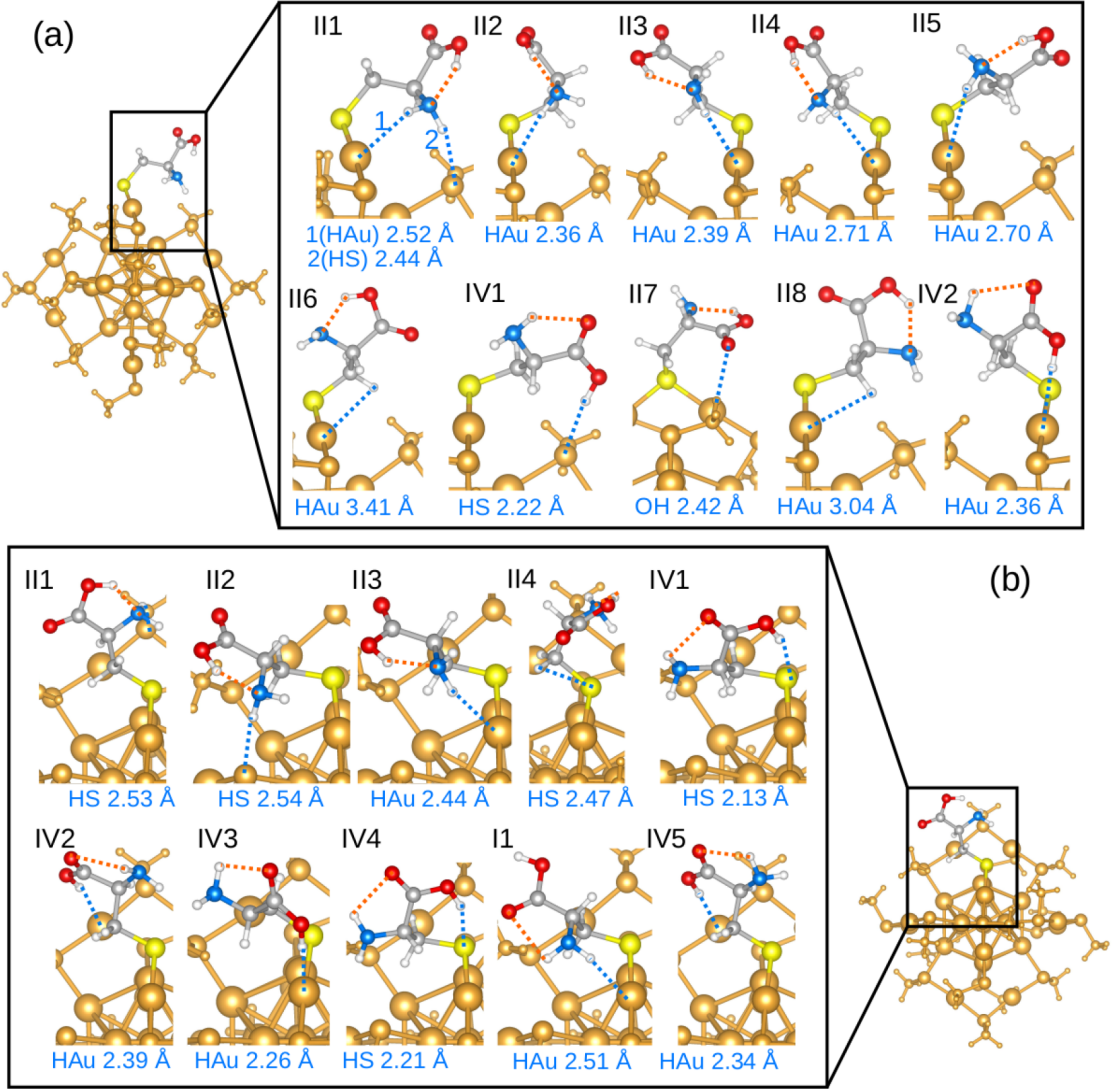
Predicted top ten low-energy conformers of system A (a) and system B (b) from the BOSS search.

Figure 7a depicts the predicted 10 lowest-energy structures of system A. The orange dashed lines mark the hydrogen bonds, while the blue dashed lines indicate the shortest atomic distance between the cysteine and Au_25_(SCH_3_)_17_. Eight out of the ten structures have type II hydrogen bonds, and two have type IV. Although structures II1–II8 all have the same kind of hydrogen bond, the energy varies by 0.33 eV, suggesting that the interactions between the cluster and cysteine play important roles in stabilizing the systems. In the structures shown in Figure 7a, the shortest distance between cysteine and the cluster is either from a hydrogen atom in the cysteine to a gold atom in the cluster or from a hydrogen atom in cysteine to a sulfur atom in the cluster. This suggests that the week H–Au and H–S interactions help to stabilize the system. These interactions could also explain why type IV hydrogen bonds do not exist in the reference cysteine conformers but often appear in systems A and B: the trans-COOH configurations facilitate the formations of the H–Au and H–S interactions.

Figure 7b depicts the predicted 10 lowest-energy structures of system B. The four lowest-energy structures have type II hydrogen bonds followed by four structures with type IV hydrogen bonds and one with type I. Similar to system A, the H–Au and H–S interactions help to stabilize the low-energy structures in system B. However, comparing Figure 7a and b, it is clear that the local environments significantly affect the energy ranking of the cysteine conformers on the cluster.

**Figure 8:**
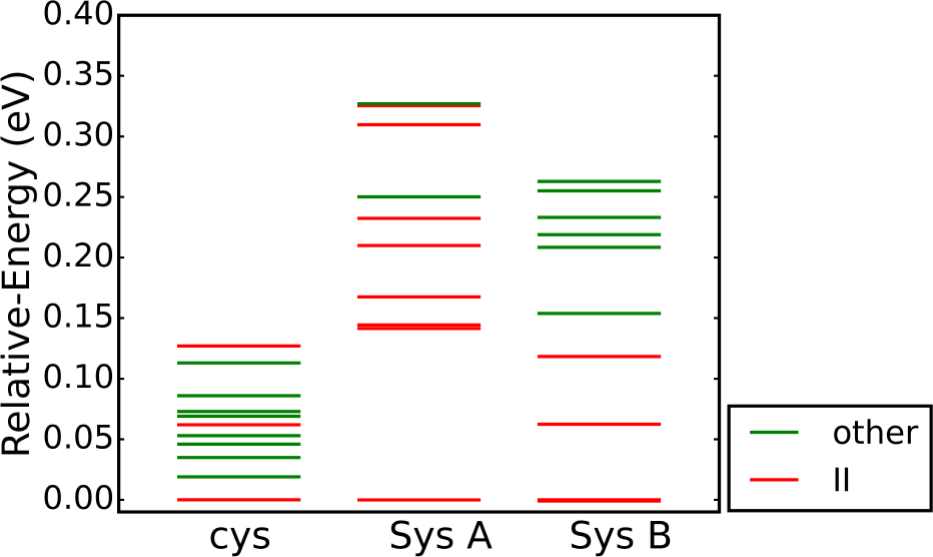
Relative energies of the ten most stable structures of cysteine molecules, system A, and system B. The red line means the structure contains a type II hydrogen bond in cysteine, while the green line means other types of hydrogen bonds in cysteine.

We plot the energies of the structures shown in Figure 7 in Figure 8 and include the energies of the top ten most stable gas-phase cysteine conformers. Comparing the energy ranking, we observe that systems A and B exhibit larger energy spacings between different configurations than the isolated cysteine molecular conformers. Second, type II hydrogen bonds are dominant in systems A and B. The structures within 0.15 eV from the global minimum of systems A and B all have type II hydrogen bonds. In contrast, for isolated cysteine, most conformers in the same energy window have other types of hydrogen bonds (types I and III). In addition, the energy difference between the lowest and the second-lowest structure is much larger in system A than in system B or in the isolated molecule. We attribute this difference to short H–S and H–Au distances we found in system A, but not in the other two.

## Conclusion

In summary, we have developed three simple strategies to handle steric clashes during the active learning. We proposed an approach that combines the strategies with BO and DFT to search for low-energy structures of a molecule on a cluster. We chose a cysteine molecule on a well-studied gold–thiolate cluster as a model system to test and demonstrate our method. Based on the tests in this work, we recommend an “energy transformation” strategy that suppresses high energies with a logarithmic transformation and applies an energy penalty to nonphysical structures where DFT computations fail. This strategy results in a smooth BOSS simulation and effective learning of the low-energy region of the PES. However, for a system whose physical and nonphysical areas in phase space can be well separated by restricting one sampling parameter, strategy **i** could be a practical choice.

For cysteine on the gold–thiolate cluster, we obtained the low-energy configurations with only 1000 single-point energy calculations and a few tens of structure optimizations. The number of DFT calculations required is similar to our previous work on gas-phase molecules, again demonstrating the high efficiency of active learning with BOSS. We found that the cysteine on the cluster inherit the conformer types of an isolated cysteine molecule. The whole system is stabilized by both internal hydrogen bonds in cysteine and the H–Au and H–S interactions between the cysteine and the gold–thiolate cluster. However, the cluster environment reorders the energies of the conformer types and enlarges the energy gaps between different configurations.

Our approach is computationally tractable and versatile. The strategies developed in this work to avoid steric clashes are not limited to BO applications. Although we chose a molecule on a cluster as a test system, our approach is suitable for other systems prone to steric clashes, such as long-chain molecules, metal–organic clusters, molecules on nanoparticles, etc. The identified molecular configurations on clusters could also be used as building blocks to construct molecule–metal hybrid clusters such as ligand protected clusters, which we will investigate in future work.

## Data Availability

The following is a list of software and websites: BOSS, https://gitlab.com/cest-group/boss; FHI-aims, https://fhi-aims.org; data for building GP models with BOSS, https://zenodo.org/record/7012914#.Y72x63bMJaQ; and coordinates of stable local minimum structures, https://nomad-lab.eu/prod/v1/gui/dataset/id/b29x8p2IRSCUtYdbeBFs9A.

## Supporting Information Available

(Figure S1) Shortest atomic pair distance between the cysteine and the cluster (*D*_*min*_) vs *d*_1_ in system A and system B. (Figure S2) *D*_*min*_ vs *d*_*i*_ (i = 2, 3, 4, 5) in system A. (Figure S3) *D*_*min*_ vs *d*_*i*_ (i = 2, 3, 4, 5) in system B. (Figure S4) Distribution of the observed energy of system A using strategy **ii** with *E*_0_ = 2.0, 4.0, 6.0 eV. (Figure S5) Distribution of the observed energy of system A using strategy **iii** with *E*_*cut*_ = 1.0, 2.0, 3.0 eV. (Figure S6) Convergence information on BOSS hyperparameters length scales for system A with strategies **i**, **ii**, and **iii**. (Figure S7) Convergence information for the global minimum of system A with strategies **i**, **ii**, and **iii**. (Figure S8) Convergence information for the global minimum of system B with strategy **iii**. (PDF)
