# Supplementary material for: Exploring the Conformers of an Organic Molecule on a Metal Cluster with Bayesian Optimization
Source: J Chem Inf Model. 2023 Jan 16;63(3):745–52. doi: 10.1021/acs.jcim.2c01120 (PMC9930108; doi:10.1021/acs.jcim.2c01120)
Supplement: Supplementary file 1 — ci2c01120_si_001.pdf [file ci2c01120_si_001.pdf]

Supporting Information

Exploring the conformers of an organic molecule on a metal cluster with Bayesian optimization

Lincan Fang,† Xiaomi Guo,‡ Milica Todorović,¶ Patrick Rinke,† and Xi Chen*,†

†*Department of Applied Physics, Aalto University, 00076 AALTO, Finland*

‡*State Key Laboratory of Low Dimensional Quantum Physics and Department of Physics, Tsinghua University, Beijing, 100084, China*

¶*Department of Mechanical and Materials Engineering, University of Turku, FI-20014 Turku, Finland*

E-mail: xi.6.chen@aalto.fi

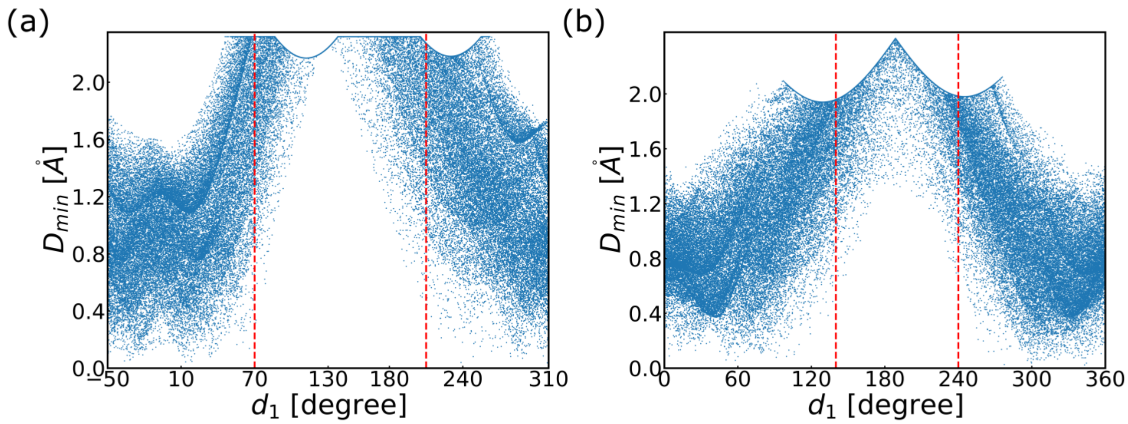

Figure S1: The shortest atomic pair distance between the cysteine and the $Au_{25}(SCH_3)_{17}$ cluster (D_{min}) vs. d_1 in (a) system A and (b) system B. The restricted sampling region of d_1 in strategy **i** is between the two red dashed lines.

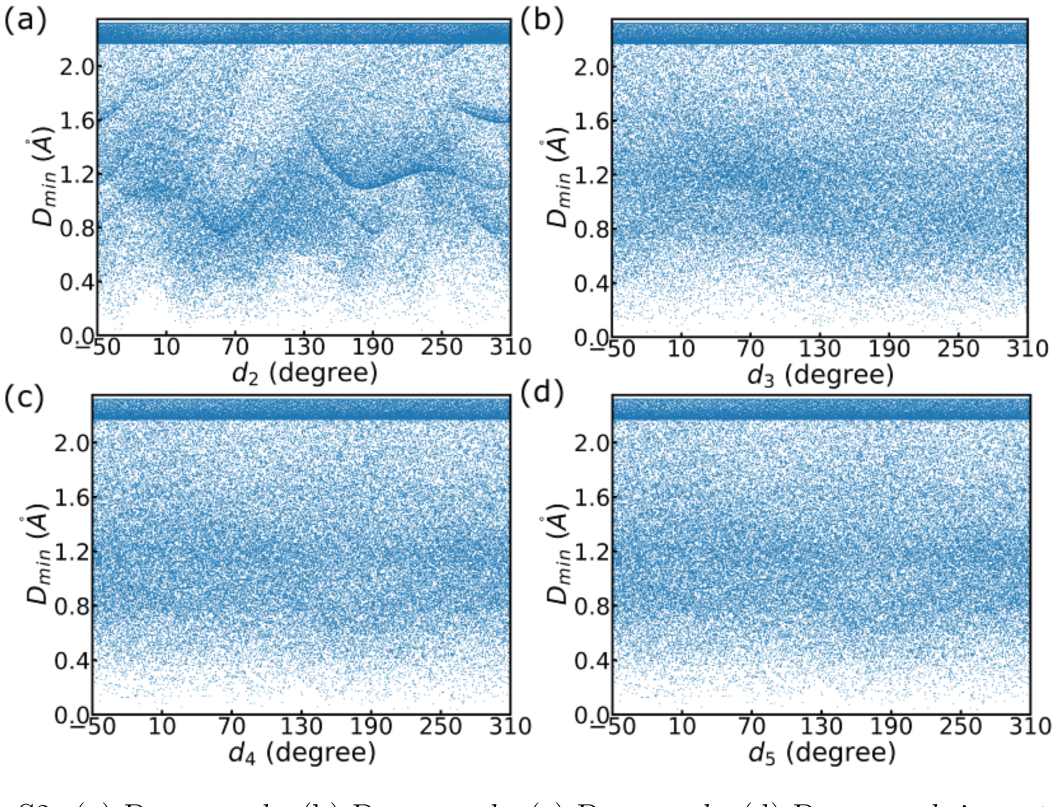

Figure S2: (a) D_{min} vs. d_2; (b) D_{min} vs. d_3; (c) D_{min} vs d_4; (d) D_{min} vs. d_5 in system A.

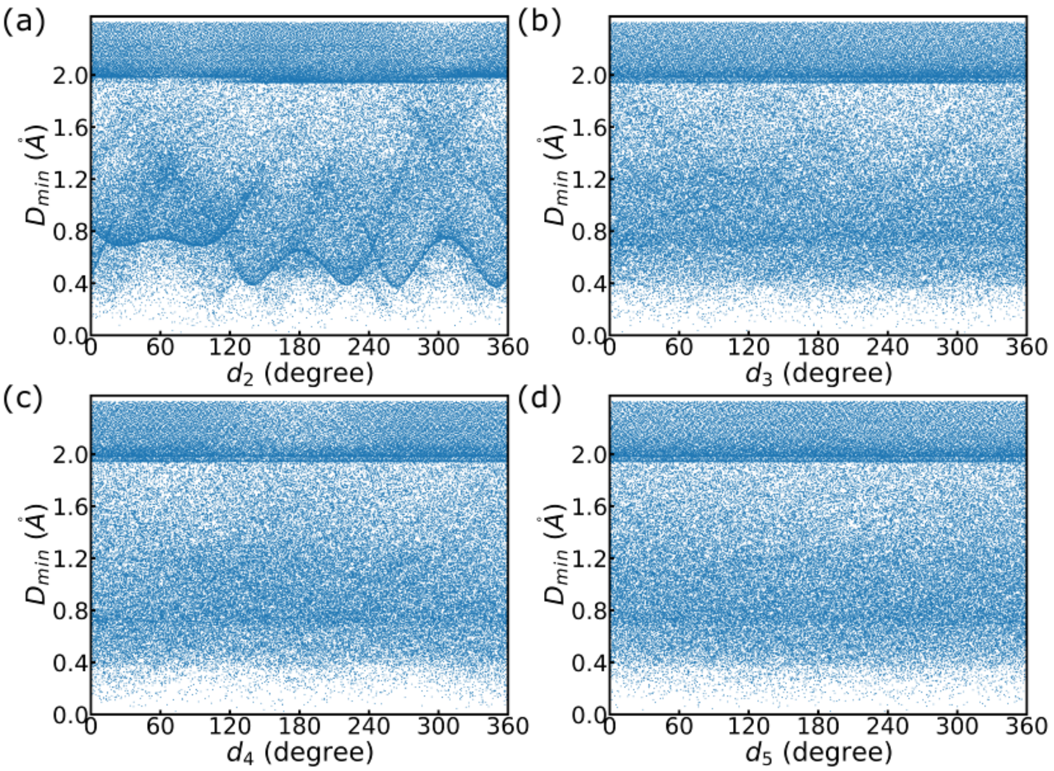

Figure S3: (a) D$_{min}$ vs. d_2; (b) D$_{min}$ vs. d_3; (c) D$_{min}$ vs d_4; (d) D$_{min}$ vs. d_5 in system B.

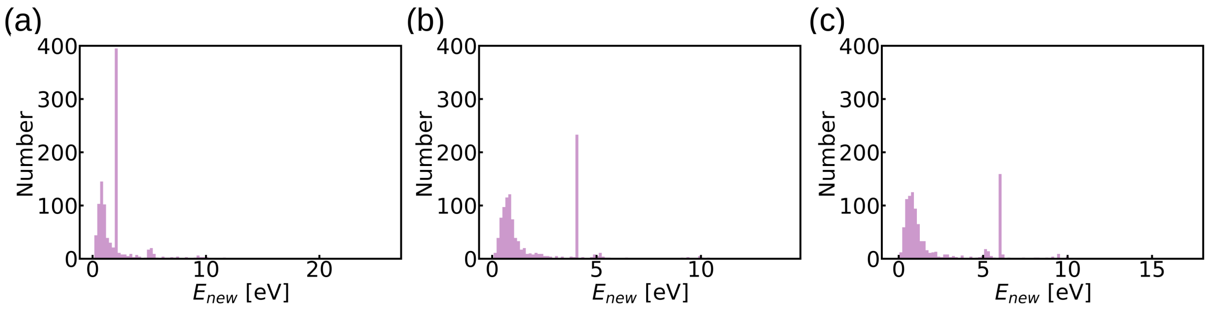

Figure S4: The distribution of the E_{new} of system A using strategy **ii** with (a) $E_0 = 2$ eV; (b) $E_0 = 4$ eV; (c) $E_0 = 6$ eV.

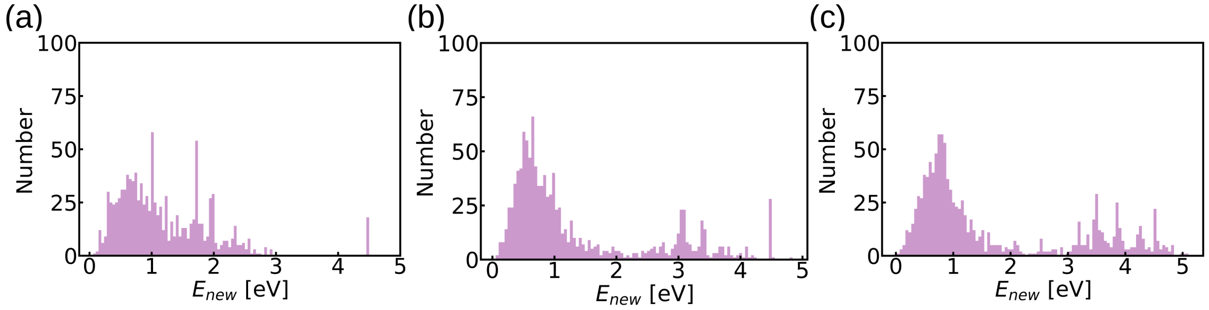

Figure S5: The distribution of the E_{new} of system A using strategy **iii** with (a) $E_{cut} = 1$ eV; (b) $E_{cut} = 2$ eV; (c) $E_{cut} = 3$ eV.

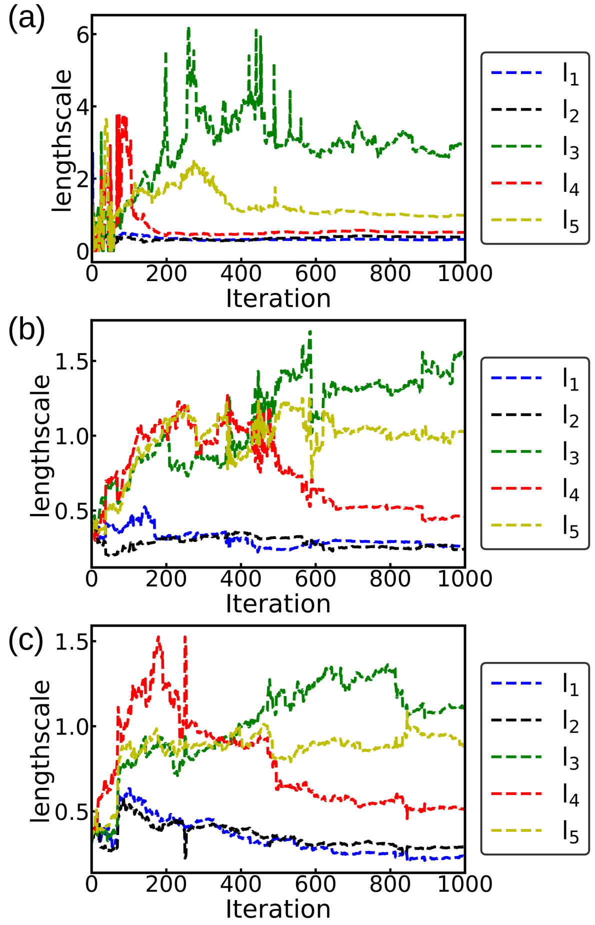

Figure S6: The convergence information of BOSS hype-parameters lengthscales for system A with (a) strategy **i**, (b) strategy **ii** and (c) strategy **iii**.

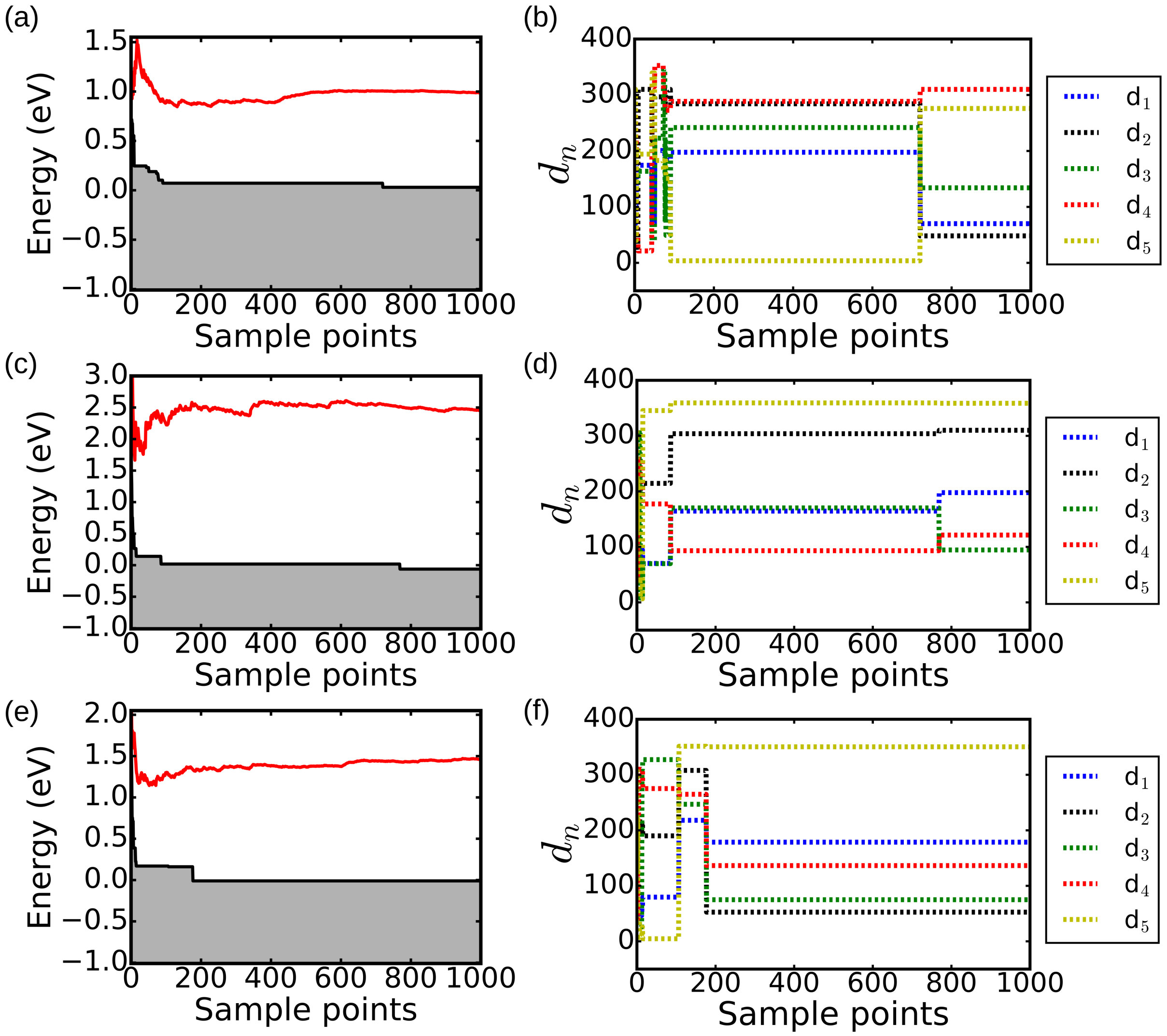

Figure S7: The convergence information for the global minimum of system A with strategy **i**: (a) Convergence of the global minimum energy computed from the BOSS-predicted global minimum configuration (black line). The average computed energy of the sampled conformers is shown with a red dashed line. (b) Value of the dihedral angles d_i (i= 1, 2, 3, 4, 5) of the BOSS-predicted global minimum as a function of the number of sampled points. The same information for strategy **ii** and **ii** is shown in (c), (d) and (e), (f).

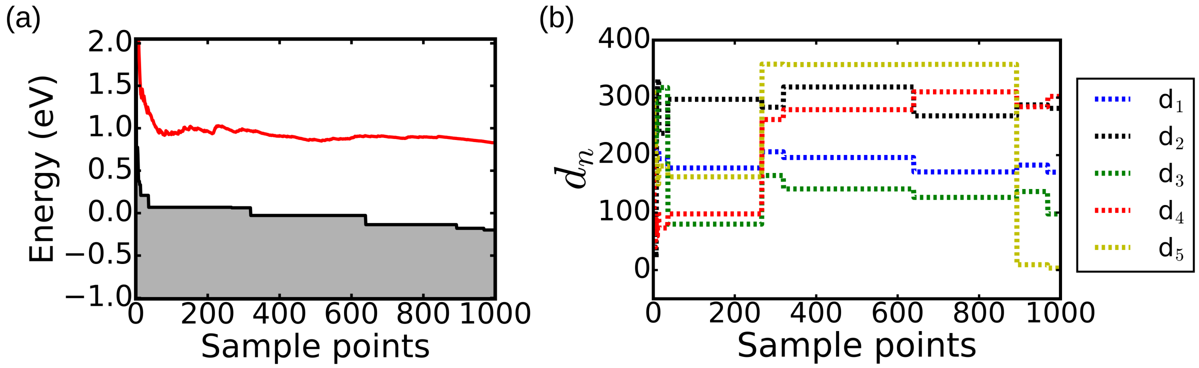

Figure S8: The convergence information for the global minimum of system B with strategy **iii**: (a) convergence of the global minimum energy of computed from the BOSS predicted global minimum configuration (black line). The average computed energy pf the sampled conformers is shown with red line. (b) Value of the dihedral angles d_n of the BOSS predicted global minimum as a function of the sampled points.
